# Using Metal-Multilayer-Dielectric Structure to Increase Sensitivity of Surface Plasmon Resonance Sensor

**DOI:** 10.1186/s11671-017-2073-1

**Published:** 2017-04-24

**Authors:** Svitlana G. Ilchenko, Ruslan A. Lymarenko, Victor B. Taranenko

**Affiliations:** International Center “Institute of Applied Optics”, National Academy of Science of Ukraine, 10G Kudryavska street, Kyiv, 04053 Ukraine

**Keywords:** Surface plasmon resonance, Multilayer metal-dielectric structure, Sensitivity of sensor

## Abstract

We propose using a specially designed metal-multilayer-dielectric structure deposited on glass substrate to enhance the evanescent field and improve the sensitivity of the surface plasmon resonance sensor. The proposed structure supports both hybrid plasmonic transverse magnetic modes and conventional waveguide transverse electric modes. We show numerically the significant enhancement of the evanescent field and improvement of the sensitivity for the waveguide transverse electric mode.

## Background

Surface plasmon resonance (SPR)-based structures have already proved their applicability as sensors, because they can rather accurately measure changes in the refractive index which occurs near the metal film surface where the evanescent field is located [1–3]. One of the possible ways to determine the SPR conditions is the analysis of dependency of the reflected light intensity on the angle of the incident light beam or its wavelength. The change of refractive index of the material to be measured leads to the change in the resonance conditions. Measuring the dynamic response of SPR, one can determine characteristics of the material in real time. The most widely used plasmonic sensor structure consists of only one thin metal film deposited on the base of a glass prism (Kretschmann configuration) for which the surface plasmon polariton (SPP) is excited by the transverse magnetic (TM) light beam and the evanescent field is formed at the vicinity of metal film surface. Small changes in refractive index near metal surface result in a shift of the resonance dip. But the SPP has quite high propagation loss, and therefore, the resonance dip has a large width which limits the resolution and sensitivity of plasmonic sensors [4–7]. When a dielectric layer is incorporated onto the metal film, then a waveguide structure is created which supports both the plasmonic mode and the conventional waveguide modes [8]. Due to narrower waveguide resonances in comparison with the SPR, this structure overcomes some of the limitations of plasmonic sensors. However, to realize the highest sensitivity of this sensor, the nanometer-sized holes should be created in the waveguide for maximization of the overlap of the zero-order waveguide mode and the material to be detected.

In this work, we consider a modified type of the plasmonic waveguide sensor [9–11] having metal-multilayer-dielectric (MMD) structure [12] which supports both hybrid plasmonic TM modes [9] and conventional waveguide transverse electric (TE) modes. The MMD structure is specially designed to further decrease the propagation losses and enhance the evanescent field for the TE mode, thereby increasing the resolution and sensitivity of the sensor.

## Methods

**Fig. 1:**
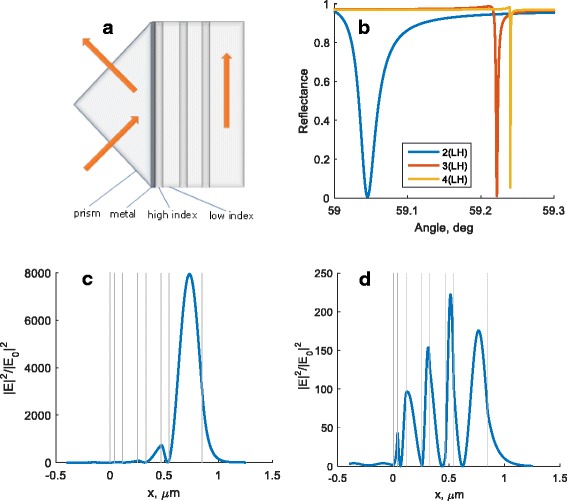
MMD structure (**a**). Reflectance of MMD structure with two, three, and four pairs of HL dielectric layers (**b**). Distribution of light intensity inside MMD structure at 532-nm wavelength under resonance conditions for TE waveguide mode (**c**) and TM hybrid plasmonic mode (**d**). *Vertical gray lines* represent MMD structure interfaces

We consider the MMD structure consisting of the silver (Ag) film of 43 nm thickness and three pairs of high and low refractive index layers (Fig. 1a). As a high index layer (H), we consider TiO_2_ (*n* = 26,678 on wavelength λ = 532 nm) and as a low index layer—SiO_2_ (*n* = 14,607 at λ = 532 nm). The core of this structure operation is based on the phenomenon of total internal reflection. The calculations are performed with the S-matrix method [13]. We found the optimal thicknesses of the layers, which provide the maximum of the field enhancement [14] in the last dielectric layer.

The structure can contain two, three, or four pairs of HL dielectric layers. The thicknesses *h* of dielectric layers can be determined from the formula ${h}_i=\frac{\lambda}{4\sqrt{\varepsilon_i}}\frac{\alpha}{ \cos \left(\varphi \right)}$, where *ε*_*i*_ is dielectric permittivity of *i* ^*th*^ layer and is a wavelength, and *α* and *φ* represent scale factors for last layer and whole structure, respectively. The optimization of the layer width leads to the following scale factors: φ = 49° and α = 1, for the last layer α = 2.2.

Figure 1c demonstrates the electromagnetic field enhancement for TE-polarization in the last dielectric layer. The amplitude of electric field for TM-polarization is significantly less than for TE-polarization. For the calculations, we chose wavelength of 532 nm. Resonant angle for 4(LH) structure is 59.22° for TE-polarization and 48.30° for TM-polarization. There are two hybrid plasmonic modes for TM-polarization at different resonant angles 48.30° and 61.9°. The structure coefficient α for the last dielectric layer differs from the unity and lies in the range from 1.5 to 2.5.

## Results and Discussion

The optimization for the thicknesses of the layers was held by variation of the parameters α and ϕ. The criterion of optimization is the maximum enhancement of the evanescent field or maximum of sensitivity for refractive index variations. For the practical applications, the presence of zero intensity in the reflected light dip center under the angle of measurement is the complementary requirement to determine the optimal layer’s thickness.

The optimal thickness of silver layer in this configuration is 43 nm. The SPR is absent for the TE-polarization of light, and formation of the waveguide modes play a key role in this case [15]. The presence of metal layer leads to the narrowing of intensity dip and significant increase of the sensor sensitivity compared to the pure SPR case or dielectric structures (without metal layer). The essence of this phenomenon is the enhancement of selective properties of the structure due to the nanosize metal layer. The combination of SPR and near half-wavelength resonators produces a new feature—the possibility of spectral selectivity handling. This opens the opportunity for practical applications of the proposed hybrid structures not only for the improvement of the sensor sensitivity but also for the development of the intracavity laser selectors [16].

In our model, we used the modified algorithm of the S-matrix method, which allows eliminating the errors and singularities [4]. The effect of essential field enhancement is also described in [15] and [16].

The peculiarity of this structure is the simultaneous observation of resonances in both TE- and TM-polarizations at different angles. The resonance in TE-polarization is very narrow (about 0.01°). For comparison, the width of resonance for TM-polarization is about 1°.

**Fig. 2:**
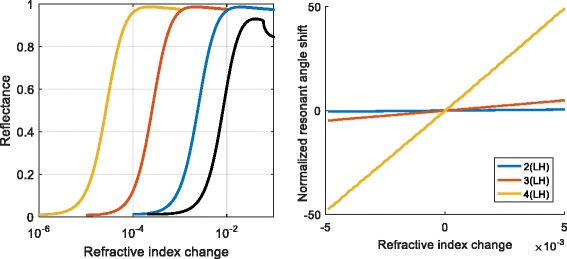
Dependence of reflectivity on refractive index at fixed angle of incidence (*left*). Dependencies of resonant angle shift normalized to dip width on refractive index (*right*)

We have compared the sensitivities of the proposed structure for TE- and TM-polarized light with Kretschmann structure (without dielectric layers) as it is shown in Fig. 2. The multilayer dielectric structure with metal layer allows increasing the sensitivity [17] up to two orders of magnitude.

Figure 2 demonstrates sensitivity of the sensor based on the MMD structures with two, three, and four pairs of LH layers. The change of reflection coefficient depending on the refractive index at fixed angle of incidence is shown in Fig. 2 (left). Note that the black curve in Fig. 2 is used as a reference and it corresponds to the Kretschmann configuration. Figure 2 (right) shows the dependencies of resonant angle shift normalized to dip width on the refractive index. The normalization is needed since the accuracy of the resonant angle shift measurement depends on the dip width. Increased number of LH pairs leads to increase of the evanescent field and narrowing of the dip width.

An important issue for the practical application of the proposed structure is the impact of layers manufacturing precision. The optimization of layer thicknesses gives the required manufacturing accuracy ±5 nm, what is technically realizable.

**Fig. 3:**
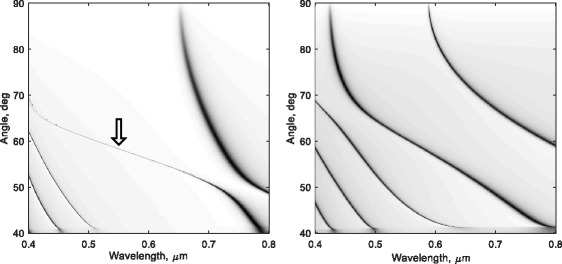
Relationship between incident angles and wavelengths for TE waveguide modes (*left*) and TM hybrid plasmonic modes (*right*). *Arrow* indicates the narrowest waveguide mode

It should also be noted that the MMD structure can operate in a wide range of wavelengths. The narrowest TE waveguide modes resonance, indicated with arrow on Fig. 3, covers practically all visible range.

## Conclusions

We have proposed a multilayer sensing structure possessing higher sensitivity compared with conventional SPR sensors. The structure combines the thin metal film and high-low dielectric layers. It supports both hybrid plasmonic transverse magnetic modes and conventional waveguide transverse electric modes. Optimal thicknesses of layers provide the maximal enhancement of the evanescent field and the highest sensitivity for the waveguide transverse electric mode.

## Abbreviations

MMD: Metal-multilayer-dielectric
SPP: Surface plasmon polariton
SPR: Surface plasmon resonance
TE: Transverse electric
TM: Transverse magnetic
